# Nanoscale Flexing Mechanism of a Metal–Organic Framework Determined by Atomic Force Microscopy

**DOI:** 10.1021/jacs.5c02868

**Published:** 2025-05-09

**Authors:** Mollie Trueman, Rachel J. S. Pooley, A. R. Bonity J. Lutton-Gething, Avantika Hasija, George F. S. Whitehead, Sean J. O’Shea, Michael W. Anderson, Martin P. Attfield

**Affiliations:** † Department of Chemistry, School of Natural Sciences, 5292 The University of Manchester, Oxford Road, Manchester M13 9PL, U.K.; ‡ Agency for Science Technology and Research, Institute of Materials Research and Engineering, Singapore 138634, Singapore

## Abstract

Flexible metal–organic frameworks (MOFs) are a unique set of compounds with applications in diverse areas. The nanoscale mechanism through which they flex is unproven. Herein, we use *in situ* atomic force microscopy to observe the crystal surface of Ga-MIL-53 MOF, [Ga­(OH)­(BDC)] (**1**) (BDC - 1, 4-benzenedicarboxylate) as it undergoes flexing transformations during the guest exchange between N,N-dimethylformamide (DMF) and ethanol (EtOH)-containing **1**. **1**·0.96DMF undergoes a flexing expansion transformation on guest exchange to form **1**·*x*EtOH through the passage of wavefronts of cooperatively transforming, consecutive rows of unit cells parallel to the (011) plane, resulting in whole (011) layers of unit cells transforming by a layer-by-layer shear mechanism. The reverse process involves **1**·*x*EtOH undergoing a flexing contraction transformation on guest exchange to form **1**·0.96DMF through a layer-by-layer shear mechanism involving layers of unit cells parallel to the (011̅) plane transforming in a cooperative manner. This proves a nanoscale mechanism through which a MOF can flex and the coexistence of phases with different degrees of expansion within a crystal, thus providing a missing link in the multilength scale understanding of MOF flexing transformations, which will support future design and application of flexible MOFs and other extended crystalline solids.

## Graphical abstract

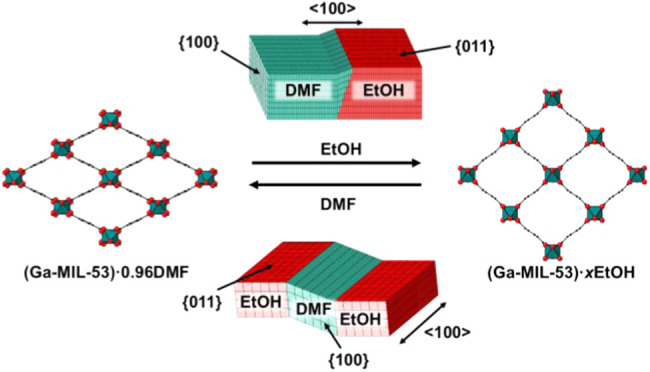

## Introduction

Porous metal–organic frameworks (MOFs) constitute a diverse class of materials that have attracted increasing interest for wide-ranging commercial applications in storage and separation processes.^1–3^ A captivating subset of the MOF family are flexible MOFs that exhibit remarkable structural flexibility by undergoing reversible transformations in response to external stimuli such as temperature, pressure, or interactions with guest species.^4–7^ These flexible compounds consist of a variety of chemical compositions and structure types. The flexing process produces unique sorption behaviors that can enhance MOF application in the storage, separation, and sensing fields.^8^ Surprisingly, the nanoscale mechanism through which the MOF crystallites flex remains unclear. In this work, we demonstrate that *in situ* atomic force microscopy (AFM) can be used to directly observe and determine a nanoscale mechanism through which a MOF crystal flexes.

The archetypal family of flexible MOF is the MIL-53 family^9^ [M­(OH)­(BDC)] (where M = Cr,^10,11^ Al,^12^ Ga,^13,14^ V,^15^ Fe,^16^ Sc,^17^ and BDC = 1, 4-benzenedicarboxylate). These MOFs are constructed from octahedrally coordinated trivalent MO_4_(OH)_2_ centers connected by μ_2_-(OH)^−^ anions into one-dimensional chains as shown in Figure 1a. The chains are connected by BDC linkers to form a ‘wine-rack’ framework containing one-dimensional pores as shown in Figure 1b. The framework undergoes reversible flexing or ‘breathing’ transformations between structures with differing degrees of expansion, as shown for the narrow pore to large pore transformation in Figure 1b.^9,12^ Flexible MIL-53 type frameworks are also made with a variety of other dicarboxylate-based linkers.^18,19^

**Figure 1:**
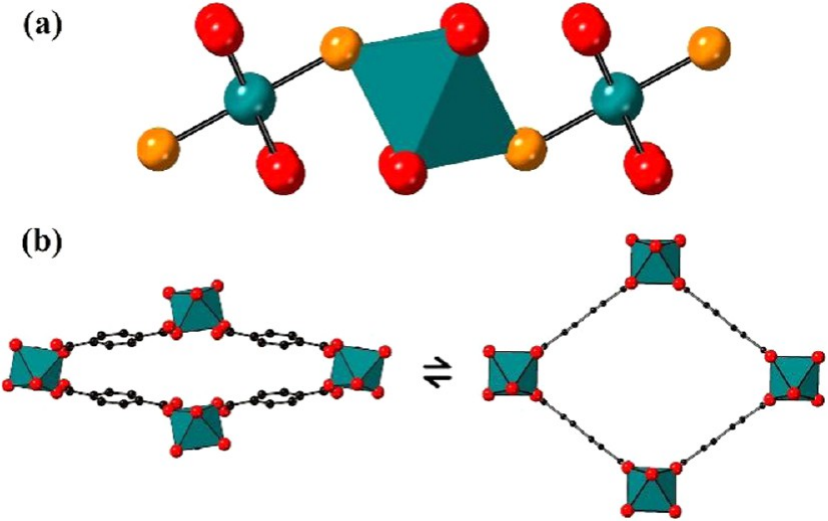
(a) Structure of a MO_4_(OH)_2_ chain of corner-sharing octahedra. (b) Schematic of a transforming narrow pore (left) to a large pore (right) MIL-53 phase. Key: dark green: M atom or M-centered octahedron, red: O, orange: O atom of μ_2_-(OH)^−^, black: C, and H atoms are omitted for clarity.

Insight into the MIL-53 flexing mechanism has been provided through *in situ* diffraction^20–24^ and electron microscopy studies,^25–27^ which have yielded essential crystal structure information from different points of the transformation, and particle geometric aspects during flexing. Spectroscopic and calorimetric studies have also identified changes in atomic coordination environments,^28^ specific functional groups,^13,29,30^ and enthalpic changes during the flexing process.^29,31^ Computer simulations have furthered understanding of the flexing process,^32–34^ predicting transformations to occur through either a layer-by-layer shear mechanism,^35,36^ or through nucleation and growth of the domains of transformed structure.^36^ Despite the strong interest in the flexing behavior of MIL-53, understanding how individual crystallites transform at the nanoscale remains limited. Currently, there are no *in situ* studies at the required spatiotemporal resolution to directly prove the flexing mechanism and the coexistence of phases with different degrees of expansion within a crystal of MIL-53 or any other coordination polymer. Such an understanding is crucial for enhancing the performance of flexible MOFs and developing novel functional MOFs and flexible extended solids.

AFM is an ideal technique to gain high temporal and nanoscopic spatially resolved images of crystal surfaces in real time under a variety of conditions.^37^ It is particularly useful for studying transformations involving MOFs due to the size of the framework organic linker and inorganic components, and that many of these processes occur under ambient conditions.^38^ AFM has been used to determine the crystal growth mechanism of several MOFs and to quantify flexible surface behavior in an MOF.^39–42^

In this work, we apply *in situ* AFM to observe, for the first time, the flexing phase transformations of a MOF at the nanoscale, thus determining that a MIL-53 MOF flexes through a layer-by-layer shear mechanism and proving the coexistence of phases with different degrees of expansion within one crystal. This provides a vital missing link in the multilength scale understanding of MOF flexing behavior.

## Results and Discussion

Single crystals of the gallium analogue of MIL-53, Ga-MIL-53, [Ga­(OH)­(BDC)] (**1**),^13,14^ with pore-enclosed guest H_2_BDC (**1**·0.74H_2_BDC) were prepared hydrothermally following a previously reported method as described in the Supporting Information (SI).^43^ Single crystal X-ray diffraction structure solution of the framework of **1**·0.74H_2_BDC and face indexing allowed determination of the orientation of the crystal structure relative to the crystal morphology. This indicated that the large expressed crystal faces are the {011} facets with the parallel <100> directions and pore direction running along the long axis of the crystal, as shown in Figure 2a. Scanning electron microscopy (SEM) imaging (Figures 2b and S1) revealed that a significant proportion of crystals had a similar morphology, allowing the large expressed facets to be identified as {011} facets on which the crystals tend to lie. This crystal orientation was assumed for the AFM studies described (*vide infra*) for crystals of similar morphology.

**Figure 2:**
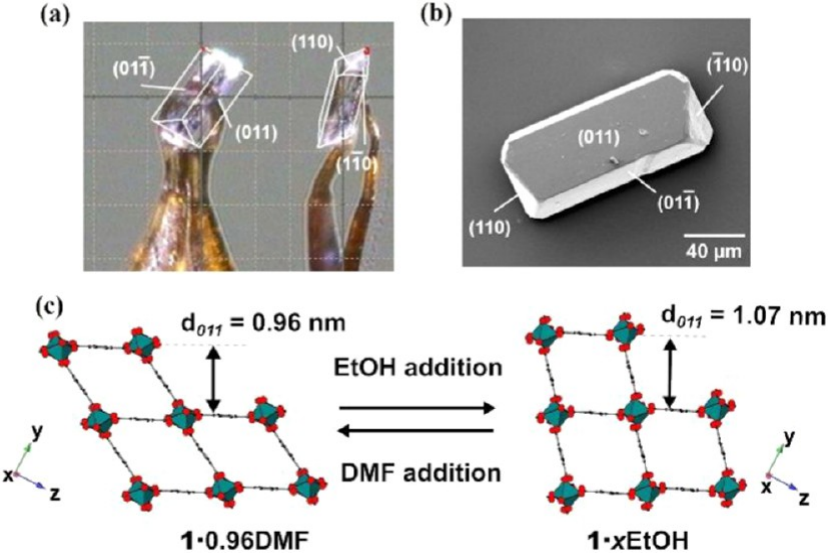
(a) Optical image of a face-indexed crystal of **1**·0.74H_2_BDC. (b) Scanning electron micrograph of a crystal of **1**·0.74H_2_BDC. (c) Schematic representation of the transformation between **1**·0.96DMF and 1·*x*EtOH.

### Guest Exchange Transformations of **1**

Solvothermal guest exchange was used to replace the H_2_BDC in the pores of **1**·0.74H_2_BDC for *N*,*N*-dimethylformamide (DMF) to form crystals of **1**·0.96DMF as determined by thermogravimetric and elemental analysis (see SI and Figures S1, S2). The crystal parameters of **1**·0.96DMF (monoclinic, *I*2 */a*, *a* = 6.7114(5) Å, *b* = 11.4123(4) Å, *c* = 17.89(1) Å, *b* = 92.310(9)°, *V* = 1368.8(1) Å^3^) determined from the Le Bail fit to the powder X-ray diffraction (PXRD) data (see Figure S3) agree well with those determined for the crystal structure of this compound.^14^ Subsequent room temperature guest exchange of **1**·0.96DMF with ethanol (EtOH) yielded previously unreported **1**·*x*EtOH with an accompanying flexing expansion transformation as indicated by the crystal parameters of **1**·*x*EtOH (orthorhombic, *Imcm*, *a* = 6.7429(2) Å, *b* = 14.3925(4) Å, *c* = 15.9254(4) Å, *V* = 1545.51(8) Å^3^) determined from the Rietveld refinement of **1**·*x*EtOH (see Figure S4). The flexing transformations between **1**·0.96DMF and **1**·*x*EtOH are depicted in Figure 2c and the framework structures of **1**·0.96DMF and **1**·*x*EtOH are additionally shown in Figure S5. These flexing transformations are accompanied by a 12.90(1) % volume change relative to **1**·0.96DMF.

### *In Situ* Observation of Guest Exchange Transformations of **1**

Crystals of **1**·0.96DMF were adhered to a glass slide in an AFM fluid cell containing DMF as described in the SI. Initial micrographs of **1**·0.96DMF revealed that {011} facets are terminated by stable extended growth terraces of average height 0.99 ± 0.09 nm (see Figures 3, 4a), calculated across 100 measurements (see Figure 4b), agreeing well with the crystallographically determined 0.96 nm d_011_ spacing of **1**·0.96DMF. EtOH was then flowed through the cell at a rate of 2 mL hr^–1^. *In situ* AFM images were collected from a {011} facet during the room temperature guest exchange of **1**·0.96DMF to **1**·*x*EtOH. The micrograph series shown in Figures 3, S6, and video SV1 begins 78 min after the introduction of EtOH at an EtOH concentration of *ca*. 93% v/v. No indications of transformation were observed prior to this time. The micrographs in Figures 3, S6, and video SV1 reveal the progression across the crystal surface of two wavefronts that travel in opposite <100> directions. The wavefronts emerge from opposite ends of the image and presumably emanate from where EtOH first enters the pores at the ends of the crystal (as seen in the optical image of the crystal in Figure 3h), a crystal domain, or through a defect such as that seen in Figure 3a. The behavior of these wavefronts is also shown in Figure 4c, revealing the wavefronts forming, traversing the surface, and merging to form a wave-free surface. The origin of the surface uplift within the wavefront is the flexing expansion transformation of the local structure of **1**·0.96DMF to **1**·*x*EtOH as the wavefront progresses and EtOH passes through the pores in the <100> directions. This is further verified by extensive measurements of the stable extended growth terraces of a {011} facet of newly formed **1**·*x*EtOH that yield an average height of 1.07 ± 0.10 nm (see Figures 4a,b), agreeing well with the crystallographically determined 1.07 nm d_011_ spacing of **1**·*x*EtOH. Large surface uplifts of the wavefront of *ca*. 7.6 nm over a lateral distance of *ca*. 1.0 μm (see Figure S7) suggest that the flexing transformation involves at least *ca*. 76 unit cells below the crystal surface and over *ca*. 1429 unit cells in the <100> directions. The surface uplift is less than the height of the single crystal and may reflect that only a small depth of the crystal is able to transform fully, as the remaining portion is submerged in the adhesive used to affix the crystal.

**Figure 3:**
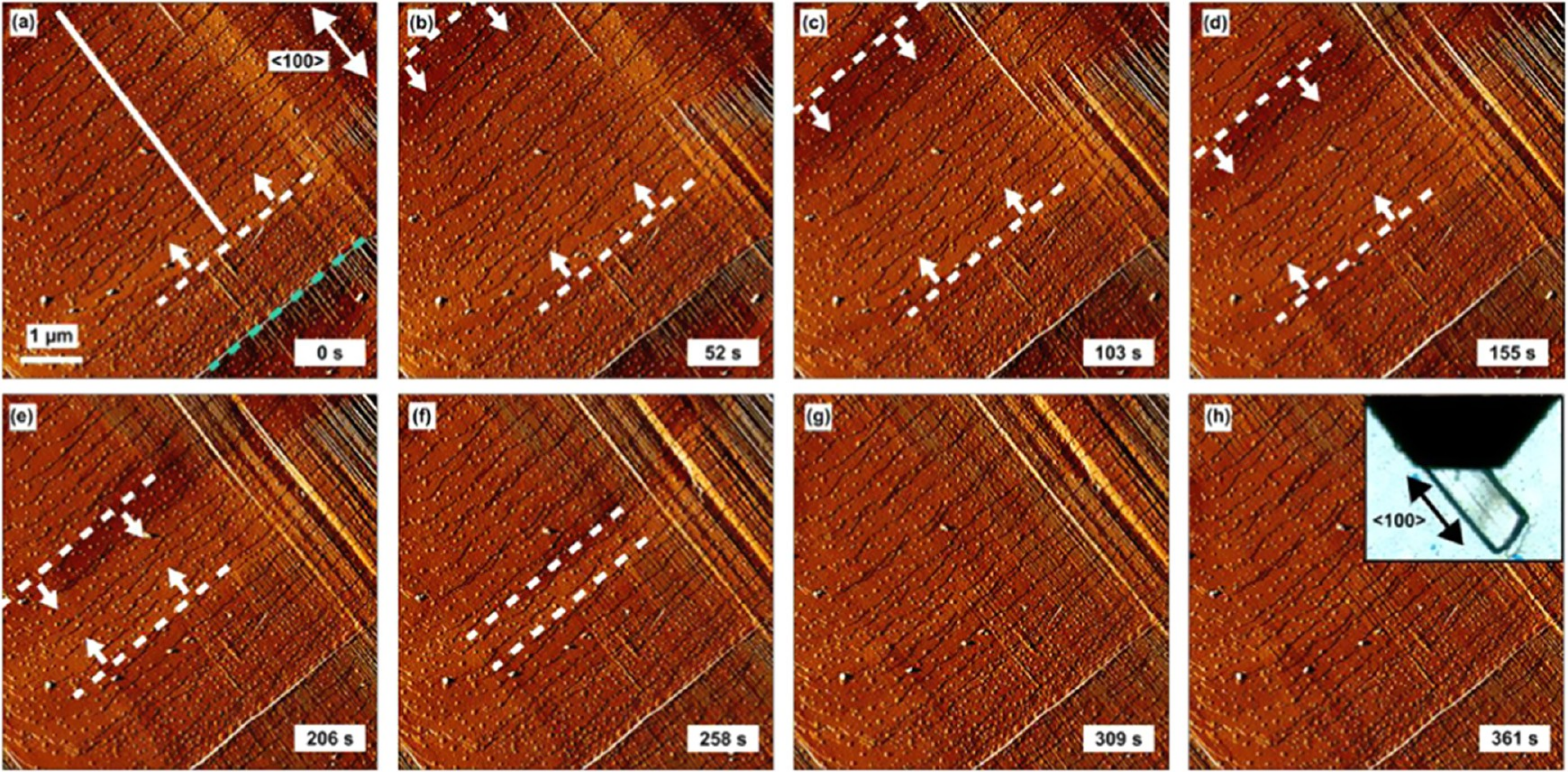
(a–h) 6.0 × 6.0 μm^2^ error signal atomic force micrographs of a {011} facet of **1** during the pore expansion transformation from **1**·0.96DMF to **1**·*x*EtOH. The white dashed lines and arrows in (a–f) indicate the positions and propagation direction of the wavefronts, respectively. The green dashed line and white line in (a) indicate a line defect and the line along which the height profiles in Figure 4c are measured, respectively. Inset in (h) shows an optical micrograph of **1**·0.96DMF prior to transformation.

**Figure 4:**
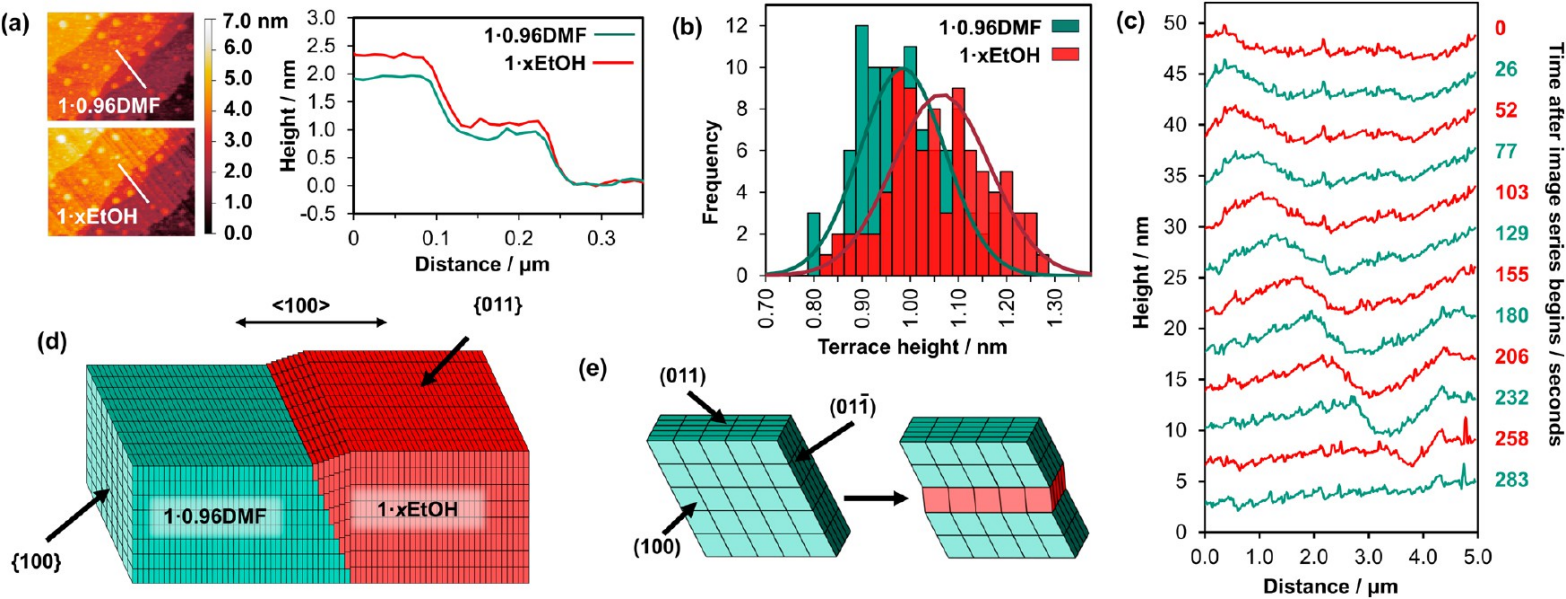
(a) Left–AFM height images showing the same 1.1 × 0.8 μm^2^ {011} region of terraces before (upper) and after (lower) the pore expansion transformation. Right–Height profiles along white lines in height images of **1**·0.96DMF and **1**·*x*EtOH. (b) Distribution of terrace heights measured from **1**·0.96DMF before the flexing expansion transformation and **1**·*x*EtOH after the transformation. (c) Temporal variation of the height profile corresponding to the white line shown in Figure 3a. (d) Schematic representation of a transforming crystal of **1**. (e) Representation of the unit cell expansion of some unit cell rows parallel to the (011) plane in **1**.

The wavefronts shown in Figures 4c and S7 are not sharp steps, suggesting that at any point during the passage of the wavefronts, the transformation has propagated in the <100> directions by varying degrees for different layers of unit cells parallel to the (011) plane, as shown in Figure 4d. This is necessary to prevent fracturing of the crystal parallel to the {100} planes, which is not observed. The transforming (011) layers shown in Figure 4d are adjacent to each other, but this is not a necessity during the crystal transformation (*vide infra*). The relatively flat nature of the wavefronts in the <011> directions suggest the flexing expansion transformation propagates much more rapidly across rows of unit cells parallel to the (011) plane in a (100) plane as shown in Figure 4e, than propagation of the transformation of unit cells in the <100> directions. This implies that a row-by-row shear mechanism is observed, where whole rows of unit cells parallel to the (011) plane cooperatively transform, which then instigates an adjacent row in the [100] direction to transform as represented in Figure 4d. The overall effect of these consecutive rows of transforming unit cells is that the whole (011) layer of unit cells transforms, indicating that a layer-by-layer shear mechanism is occurring^35,36^ over a finite time period.

The wavefronts cross the crystal surface at a constant speed of *ca*. 0.9 μm min^–1^, as shown in Figure S8a throughout the observed period. This constant speed suggests that the transformation follows Case II non-Fickian diffusion or sorption behavior, where the transformation is limited by the framework expansion rather than diffusion of the guest species through the pore structure in a manner akin to that observed for the swelling transformation of polymers.^44,45^

The surface texture transforms after wavefront passage from being relatively smooth to roughened with many closely spaced ridges running along the <100> directions as seen in Figures 3a–h and 5a–d. The height of the ridges depicted in Figure 5e, f is *ca*. 0.5 nm. The texture development may result as a mechanism to reduce bulk and surface stress and strain developed due to the volume expansion of the crystal domains being imaged relative to the surrounding domains that may transform to a different extent or at a different rate.^46^ The volume expansion of the crystal domains during the transformation arises from the combined expansion of the pores and the increase in distance between the chains of Ga^3+^-centered octahedra from 10.608(6) Å in **1**·0.96DMF to 10.7325(2) Å in **1**·*x*EtOH. The formation of the ridges and their cross-sectional shape shown in Figure 5a–f suggest that these ridges are formed by opposing shears on a few adjoining layers of unit cells parallel to the (011̅) planes, as shown in the simplified schematic for only two layers of distorted unit cells in Figure 5g. The ability of the framework to undergo such deformations is accommodated by the ability of the BDC linkers, the chains of GaO_4_(OH)_2_-centered octahedra, and connections between the latter components to distort^47–49^ and is predicted for another dicarboxylate-containing MOF subjected to compressive forces.^50^ These ridges persist on the surface of **1**·*x*EtOH.

**Figure 5:**
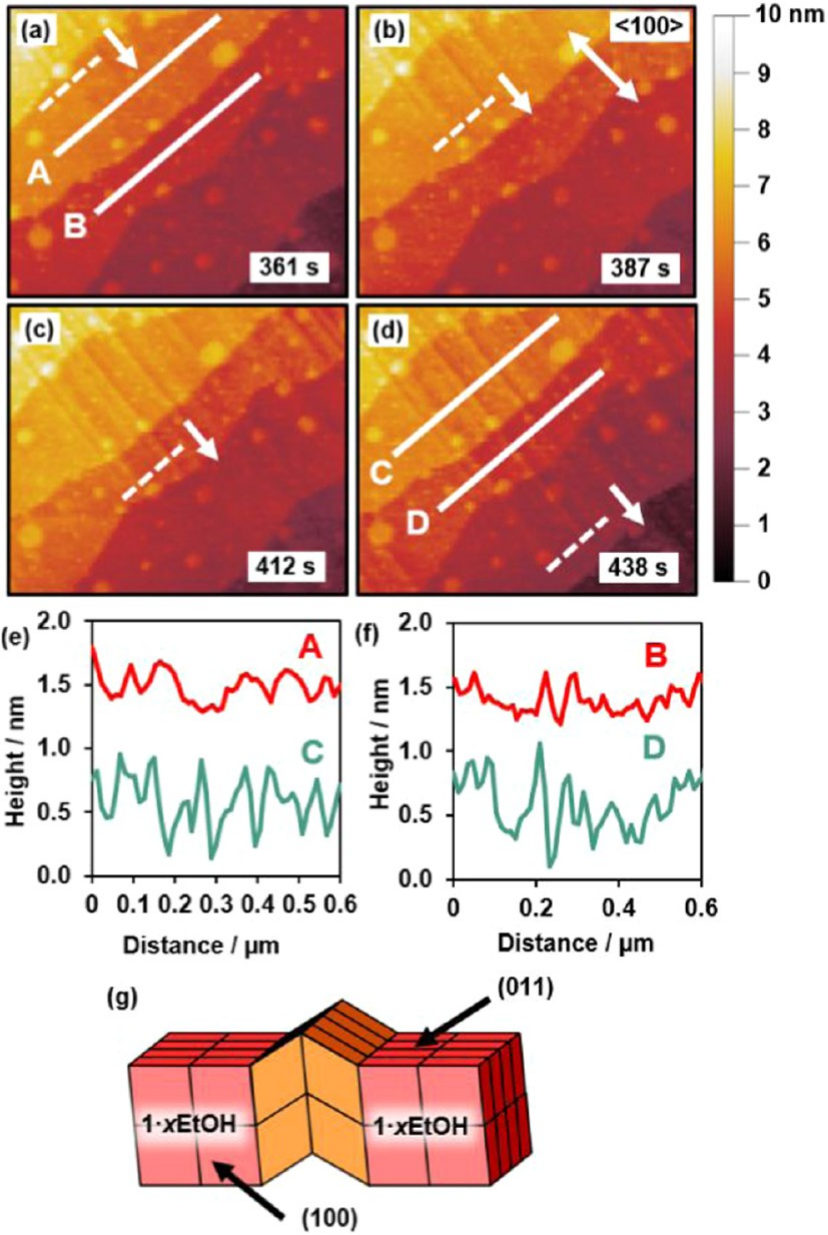
(a–d) AFM height images showing the same 0.875 × 1.25 μm^2^ {011} crystal surface region of **1**·0.96DMF as a wavefront passes through it during transformation to **1**·*x*EtOH. (e, f) Height profiles before and after transformation corresponding to white solid lines A–D in (a) and (d). (g) Schematic representation of a ridge in the crystal surface simplified to two layers of unit cells distorted relative to the geometry of the unit cell of **1**·*x*EtOH. The white dashed line and arrow in (a–d) indicate the position and propagation direction of the wavefront, respectively, and the orange unit cells in (g) are those distorted relative to **1**·*x*EtOH.

Simultaneous acquisition of optical micrographs throughout the data collection revealed crystals to darken and lose optical transparency, as shown in Figure S9. The loss of optical transparency indicates the development of cracks in the crystal during the flexing expansion transformation. Such cracks are also found in some AFM images during the flexing expansion transformation of **1**·0.96DMF (as shown in Figure S10) that run along the <100> direction and may additionally contribute to the stress and strain fields forming the closely spaced ridges running along the <100> directions. Changes in optical transparency were observed 5 min after the onset of observable changes in AFM images and continued for approximately 9 min, suggesting phase transformations initiate at the surface before interior regions transform.

MIL-53 flexing transformations are generally reversible,^9^ so the flexing transformation of **1**·*x*EtOH to **1**·0.96DMF was studied. **1**·*x*EtOH was formed by submerging affixed **1**·0.96DMF crystals in 1 mL of EtOH. Crystals of **1**·*x*EtOH were formed over a period of *ca*. 120 s as monitored by the darkening and loss of optical transparency of the crystals shown in Figure S11. A 20% v/v solution of DMF in EtOH was injected into the fluid cell at a rate of 1 mL hr^–1^. *In situ* AFM images were collected from the {011} facet during the room temperature guest exchange of **1**·*x*EtOH to **1**·0.96DMF. The first indications of changes associated with transformation were noted 1120 s after injection of the DMF solution at a DMF concentration of *ca*. 5.3% v/v in the AFM fluid cell.

The micrographs in Figures 6, S12, S13, and video SV2 reveal dark bands emerging from opposite image ends that emanate from where the DMF can first enter the pore system at the ends of the crystal (as seen in the optical image of the crystal in Figure 6h) or a crystal domain. These bands pass rapidly across the surface in the <100> directions at a constant speed of *ca*. 0.6 μm min^–1^, see Figure S8b, again suggesting that the transformation follows Case II non-Fickian diffusion or sorption behavior.^44,45^ After crossing the surface, the bands spread out in the surface <011> directions at a speed of *ca*. 8 nm min^–1^ to merge with one another. The coloration in the micrograph series in Figures 6, S13, S14, and video SV2 is based on error signal imaging. Under this coloring scheme, a darker color indicates a more negative gradient along the fast scan direction, so the dark strips in Figures 6, S13, S14, and video SV2 represent regions of the surface inclined to the original surface. This is also shown in Figure 7a, which shows regions of the surface that lie at an angle to the original surface and grow with time. Measurement from the micrographs of the angle between the surfaces highlighted in Figure 7b reveals a mean angle of 162.1° ± 1.1° determined from 100 measurements as shown in Figure 7c that offers good agreement with the value of 160.9° (95.8° + 65.1° from the internal pore angles shown in Figure S5a,b) deduced from the predicted interface of **1**·*x*EtOH and **1**·0.96DMF. The formation and spreading of these dark bands correspond to nucleation and growth of domains of **1**·0.96DMF from **1**·*x*EtOH through a flexing contraction transformation as DMF travels along the pores and proves the coexistence of **1**·*x*EtOH and **1**·0.96DMF within one crystal. The surface becomes relatively smooth after transformation, as shown in Figures S13 and S14, and appears to have maintained full surface connectivity.

**Figure 6:**
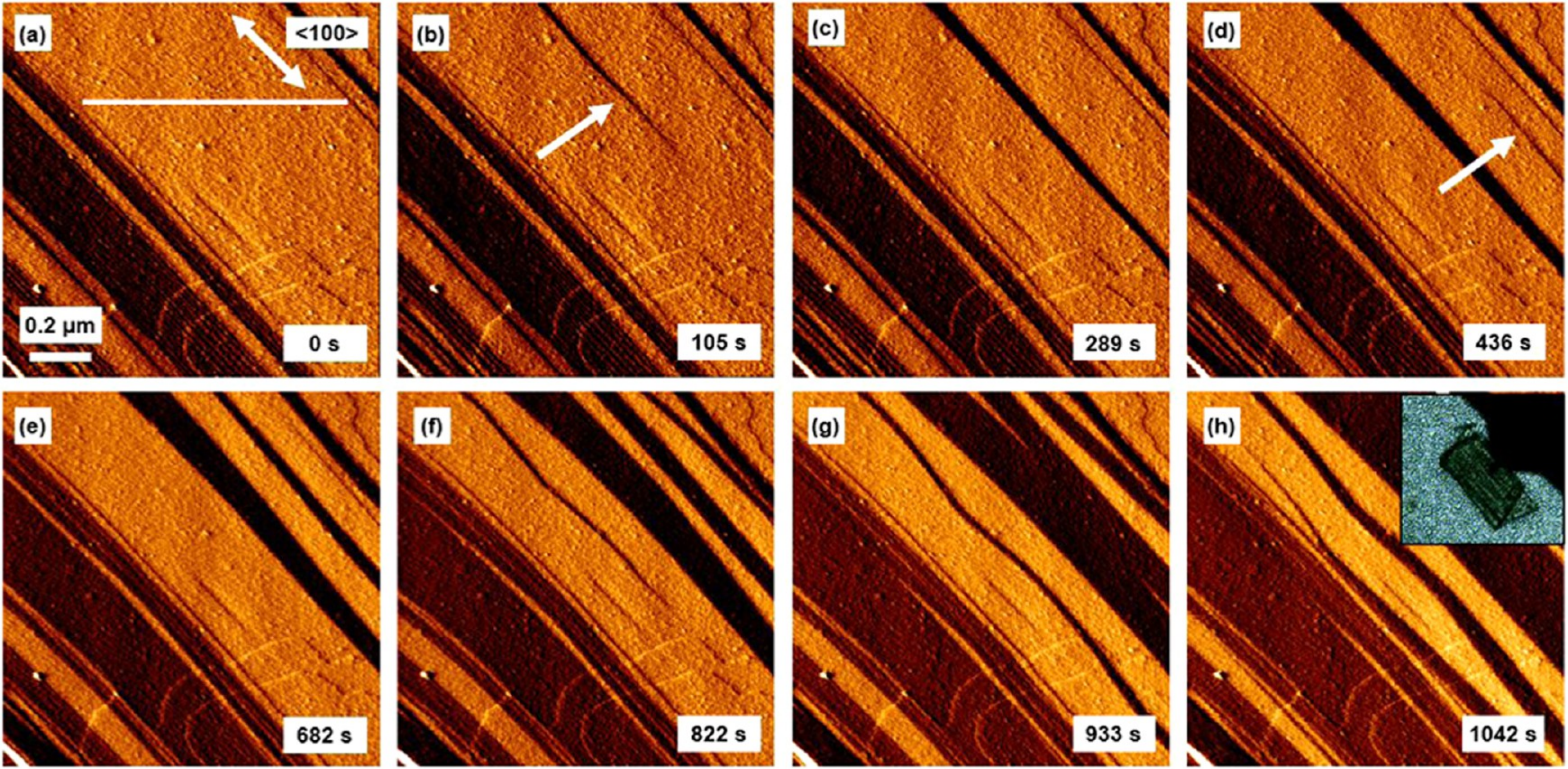
(a–h) 0.85 × 0.85 μm^2^ error signal atomic force micrographs of a {011} facet of **1** depicting the pore contraction transformation from **1**·*x*EtOH to **1**·0.96DMF with the inset in (h) showing the optical micrograph of **1**·*x*EtOH. White arrows in (b) and (d) indicate transforming parts of the crystal emanating from different image ends. The white line in (a) indicates the line along which the height profiles in Figure 7a are measured.

**Figure 7:**
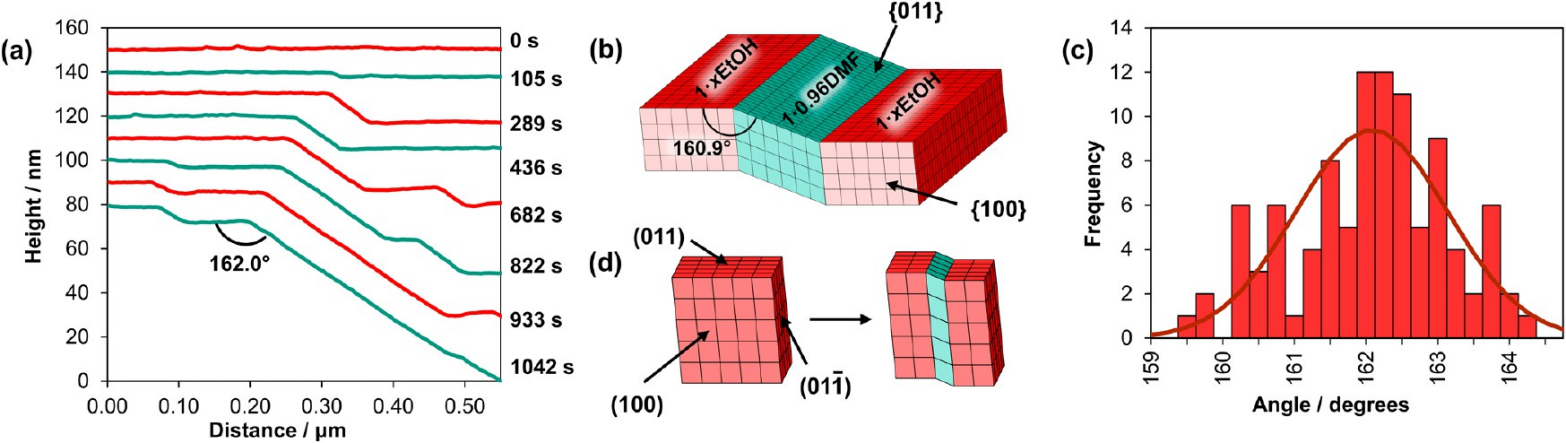
(a) Temporal variation of the height profile corresponding to the white line shown in Figure 6a. (b) Representation of the proposed structure accounting for the formation of angled regions at the surface. (c) Distribution of the angles between the surface planes measured from a {011} surface of **1**·xEtOH as it undergoes a flexing contraction transformation to **1**·0.96DMF. (d) Schematic representation of the unit cell contraction of some unit cell rows parallel to the (01**1̅**) plane in **1**.

The observed micrographs and height profiles given in Figures 6, S12–S14, and 7a suggest the mechanism of the flexing contraction transformation is a layer-by-layer shear mechanism involving whole layers of unit cells transforming in a cooperative manner parallel to the (011̅) plane as defined in Figure 7d. The layer shearing does not occur instantaneously over a whole crystal layer but propagates in the <100> directions over a finite time, presumably *via* rows of unit cells in a {100} plane transforming parallel to the (011̅) plane. Transformation of layers adjacent to the initially transforming layer is favored over transformation of new layers, as seen in Figures 6 and S13. This mechanism is largely consistent with the computationally predicted flexing contraction transformation.^35^ The **1**·*x*EtOH surface contains regions of closely spaced ridges running along the <100> directions, as seen in Figure 8a, that disappear after transformation to **1**·0.96DMF, leaving a relatively smooth surface as shown in Figure 8b. This suggests the reversible release of stress and strain induced after the **1**·0.96DMF to **1**·*x*EtOH transformation. Confirmation that the product of this flexible contraction transformation was **1**·0.96DMF was ascertained from the excellent agreement of the PXRD patterns given in Figure S15 with that of Figure S3.

**Figure 8:**
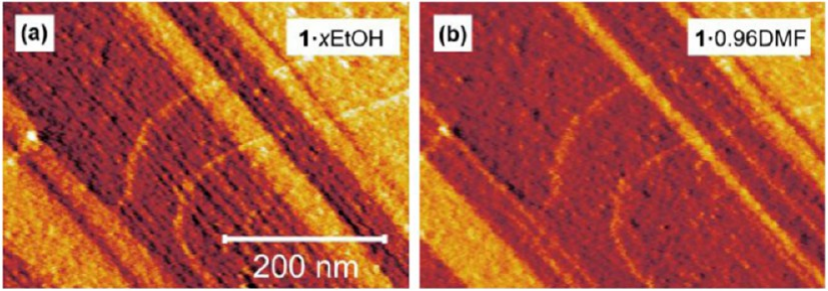
(a) Error signal atomic force micrograph showing a 0.50 × 0.35 μm^2^ {011} rippled crystal surface region of **1**·xEtOH before guest exchange. (b) Error signal atomic force micrograph showing the same region depicted in (a) following the pore contraction transformation to **1**.0.96DMF.

A combination of the flexing transformation experiments allows the observation of the shear mechanism when the induced shearing is parallel and perpendicular to the observed surface for the expansion and contraction transformations, respectively, so providing unrivaled information on the overall flexing transformation. The reasons behind the difference in shear direction for these two transformations are not immediately apparent for the crystallographically equivalent (011) and (011̅) planes. The shear direction parallel to the (011) plane for the flexing expansion transformation, as shown in Figure 4e, is most probable considering that the crystal is bound in the adhesive lying approximately flat on a (011) facet. The reason for the shear direction parallel to the (011̅) plane for the flexing contraction transformation as shown in Figure 7d is less obvious but may arise from the creation of enough defects, fractures, and interdomain microvoids during the transformation of the crystal from **1**·0.96DMF to **1**·*x*EtOH to **1**·0.96DMF to enable shearing in this direction. The transformation from **1**·0.96DMF to **1**·*x*EtOH to **1**·0.96DMF involves transformation between a monoclinic structure and an orthorhombic structure, which can be a source for the development of crystal fracture and interdomain microvoids.^51^ This is because the orthorhombic to monoclinic transition is likely to be accompanied by twinning of the crystal as reported in crystals of **1**·0.96DMF.^14^ Also, during the **1**·*x*EtOH to **1**·0.96DMF transformation, the observed crystal domains are transforming into ones of smaller volume and may transform at a different rate and to a different extent to surrounding domains, thus also potentially creating interdomain microvoids to allow shear parallel to the (011̅) plane to occur. Finally, the transformations between **1**·*x*EtOH and **1**·0.96DMF are unlikely to be completely reversible in terms of perfect realignment of crystal domains after transformation, thus creating another source of interdomain microvoids. The irreversible formation of cracks and likely domain fracturing within **1**·*x*EtOH is evident from the loss of optical transparency of the crystal of **1**·*x*EtOH compared to **1**·0.96DMF during the first expansion transformation and the maintained lack of optical transparency of the crystals after the **1**·*x*EtOH to **1**·0.96DMF transformation as seen in Figures S9, S11 and S16 respectively.

## Conclusions

This work exemplifies the power of *in situ* AFM to provide meaningful direct nanoscale mechanistic information about phase transformations within MOFs and coordination polymers. More specifically, it has been used to record the first direct observation of flexing transformations of a framework compound at the nanoscale and reveals a layer-by-layer shear mechanism through which the crystal flexes. The finite duration of the transformations has enabled the coexistence of phases with varying degrees of expansion within a crystal to be proven, potentially offering pathways to isolate crystals with distinct domains of pore openness. The results for one MOF are reported, but it is likely that the obtained mechanism is applicable to other flexible MIL-53 MOFs, their numerous derivatives, and other flexible MOFs with “wine-rack”-type frameworks. This approach should be extendable to study other flexible MOFs of different chemical composition and structure type to gain greater nanoscale insight into other possible flexing mechanisms. Such a greater fundamental understanding of the flexing transformation at the nanoscale will aid future design and application of porous MOFs and other flexible extended solids in a variety of fields.

## Supplementary Material







## Notes

Synthetic methods; experimental methods; single crystal and powder X-ray diffraction results; thermal and elemental analyses; SEM images; AFM images; optical microscopy images, and crystal structure diagrams (PDF)
Ga-MIL-53 pore expansion, DMF to EtOH guest exchange (MP4)
Ga-MIL-53 pore contraction, EtOH to DMF guest exchange (MP4)

## §

School of Chemistry, The University of Birmingham, Edgbaston, Birmingham, B15 2TT, United Kingdom
