# Supplementary material for: Nanoscale Flexing Mechanism of a Metal–Organic Framework Determined by Atomic Force Microscopy
Source: J Am Chem Soc. 2025 May 9;147(20):17201–8. doi: 10.1021/jacs.5c02868 (PMC12100706; doi:10.1021/jacs.5c02868)
Supplement: Supplementary file 1 [file ja5c02868_si_001.pdf]

# Supporting Information

# Nanoscale Flexing Mechanism of a Metal-Organic Framework Determined by Atomic Force Microscopy

Mollie Trueman,[a] Rachel J. S. Pooley,[a] A. R. Bonity J. Lutton-Gething,[a#] Avantika Hasija,[a] George F. S. Whitehead,[a] Sean J. O'Shea,[b] Michael W. Anderson[a] and Martin P. Attfield[a*]

[a] Department of Chemistry, School of Natural Sciences, The University of Manchester, M13 9PL, U.K.

[b] Institute of Materials Research and Engineering, 138634, Singapore.

[#] School of Chemistry, The University of Birmingham, Edgbaston, Birmingham, B15 2TT, U. K.

* Corresponding Author

E-mail: *m.attfield@manchester.ac.uk*

## Synthetic procedure

**Materials:** $Ga(NO_3)_3 \cdot xH_2O$ (Alfa Aesar, Puratronic, 99.999%), benzene-1,4-dicarboxylic acid ($H_2BDC$, Sigma Aldrich 98%) N,N-dimethylformamide (DMF, Fischer Scientific, 99.5%) and ethanol (Sigma Aldrich 99.8%) were used as received with no further purification. Deionised water was purified using a Milli-Q water system (18 MΩ cm resistivity at 25 °C).

**Synthesis of Ga(OH)(BDC)·0.74H$_2$BDC:** Ga(OH)(BDC)·0.74$H_2$BDC (**1**·0.74$H_2$BDC) was synthesised using the approach outlined by Vougo-Zanda *et al.*.[1] $Ga(NO_3)_3 \cdot xH_2O$ (0.263 g, 0.96 mmol for $x$ = 1) $H_2$BDC (0.343 g, 2.06 mmol) and deionised water (5 $cm^3$) were combined in a 23 ml Teflon-lined, stainless steel autoclave and heated at 220 °C for 72 hours. The resulting mixture was filtered and washed successively in deionised water (3 x 20 $cm^3$) and hot DMF (20 $cm^3$ at 65 °C) to give a white solid containing **1**·0.74$H_2$BDC alongside needles of $H_2$BDC.

**Synthesis of Ga(OH)(BDC)·0.96DMF from Ga(OH)(BDC)·0.74H$_2$BDC:** Ga(OH)(BDC)·0.96DMF (**1**·0.96DMF) was produced from **1**·0.74$H_2$BDC using the solvothermal approach taken by Chaplais *et. al.*.[2] 0.2 g of as-synthesised **1**·0.74$H_2$BDC and DMF (10 $cm^3$) were placed in a 23 ml Teflon-lined, stainless steel autoclave and heated at 160 °C for 6 days. The solid was recovered by filtration and washed in DMF (2 x 20 ml) before drying at room temperature.

**Synthesis of Ga(OH)(BDC)·*x*EtOH from Ga(OH)(BDC)·0.96DMF:** Ga(OH)(BDC)·*x*EtOH (**1**·*x*EtOH) was produced directly from **1**·0.96DMF through submersion in excess EtOH under ambient conditions.

## Characterisation and microscopy

**Single crystal X-ray diffraction:** A suitable crystal of **1**·0.74$H_2$BDC was selected and mounted on a MiTiGen polymer loop on a SuperNova, single source Eos diffractometer. The crystal was kept at 100(2) K during data collection. CrysAlisPro (v42.49) software was used to collect and reduce the data, and to refine the unit cell parameters. Using Olex2,[3] the framework structure was solved with the SHELXT[4] structure solution program using intrinsic phasing methodology and refined with the SHELXL[5] refinement package using least squares minimisation. The resulting crystal parameters (orthorhombic, P*mcn*, $a$ = 6.7614(13) Å, $b$ = 11.697(4) Å, $c$ = 17.748(3) Å, $V$ = 1403.6(6) Å$^3$) agree well with those of the previously reported crystal structure of this compound.[1] The faces of the crystal were indexed using optical images of the mounted crystal and crystal orientation operations applied using CrysAlisPro (v42.49) software.

Diffraction data were collected from a small crystal fragment of **1**·0.96DMF at 283 K as described above and used to obtain and refine the unit cell parameters (monoclinic, I2/*a*, $a$ = 6.7069(4) Å, $b$ = 11.313(1) Å, $c$ = 17.957(1) Å, $\beta$ = 91.838(6)° $V$ = 1361.8(2) Å$^3$). These unit cell parameters agree well with those reported for the crystal structure of this compound and the Le Bail fitting results (*vide infra*).[2] The crystals of **1**·0.96DMF were too small to face index.

**Scanning electron microscopy:** Scanning electron microscopy imaging was used to image crystals of **1**·0.74$H_2$BDC and **1**·0.96DMF. Images were obtained using a Q200 scanning electron microscope (SEM) operating in high vacuum mode. Samples were dispersed on a stainless-steel SEM stub and sputter coated with a thin layer of gold to an approximate thickness of 10 nm. Images were collected in secondary electron mode with a working distance of 10 mm and an accelerating voltage of 5 kV.

**Thermal Analysis:** Thermal gravimetric analysis (TGA) was conducted on **1**·0.96DMF to quantify the amount of DMF in the framework. TGA was performed on a Mettler-Toledo TGA/DSC instrument under a flow of nitrogen in the temperature range of 25°C to 600°C. The thermograph of **1**·0.96DMF, shown in Figure S2, shows two distinct mass loss events of 21.81% and 41.01%, assigned to loss of DMF and the combustion of BDC within the framework. From the 21.81% weight loss, the solvent occupancy was determined to be 0.96 DMF molecules per formula unit of framework assuming phase purity. Corresponding data could not be collected for **1**·*x*EtOH due to rapid desolvation on removal of **1**·*x*EtOH from EtOH.

**CHN analysis:** CHN analysis was conducted on **1**·0.96DMF using a Thermo Scientific Flash Smart CHN analyser fitted with a thermal conductivity detector. Measured values for **1**·0.96DMF are shown below. Calculated values are based on 0.96 formula units of DMF per unit cell. Meaningful CHN analysis could not be conducted on **1**·*x*EtOH due to rapid desolvation on removal from EtOH.

| | **Measured / wt. %** | **Calculated / wt %** |
|---|---|---|
| **C** | 40.97 | 40.71 |
| **H** | 3.61 | 3.68 |
| **N** | 4.38 | 4.19 |

**Powder X-ray Diffraction (PXRD):** PXRD data were collected for **1**·0.96DMF using a Phillips X'Pert diffractometer equipped with a Cu-Kα source. The operating current was 40 mA and the voltage was 45 kV. The sample was loaded on a zero-background, cut silicon plate. **1**·*x*EtOH was found to desolvate rapidly on removal from solution so PXRD data were collected from a sealed quartz capillary tube containing a slurry of **1**·*x*EtOH and ethanol. Data were collected on a Rigaku Oxford FR-X diffractometer equipped with a dual X-ray source and fitted with a capillary stage. Data were collected at ambient temperature and Cu-Kα radiation was used to collect data. CrysAlisPro was used to collect and process the diffraction patterns.

Le Bail fitting of the data sets for **1**·0.96DMF and **1**·*x*EtOH was performed with the final fit for **1**·0.96DMF shown in Figure S3. Starting lattice parameters and space groups were taken from the single crystal structures of the framework of **1**·0.96DMF and a large pore **1**.[2, 6] In the case of **1**·*x*EtOH, the previously reported framework structure for large pore **1**[6] was used as a starting model in a Rietveld refinement. The framework structure was refined through use of heavily restrained geometric parameters and difference Fourier electron density maps showed the presence of electron density within the void volume of the framework. This electron density was modelled through the addition of some atoms in the void volume of the framework but could not be refined as individual EtOH molecules suggesting disorder of the EtOH molecules within the void volume as has been reported for Fe-MIL-53 containing guest alcohols.[7] The final observed, calculated and difference plots for the Rietveld refinement of **1**·*x*EtOH is shown in Figure S4. Standard crystallographic data, final atomic coordinates, isotropic atomic displacement parameters and selected geometric parameters are given in the powder cif CCDC 2416167 file. The GSASII software package[8] was used to perform the Le Bail and Rietveld refinements. Values of *d*-spacings and distances between the chains of Ga-centred octahedra were calculated from the results of the Le Bail and Reitveld refinements and the locations of the Ga atoms on special positions in both structures.

**Atomic force microscopy (AFM):** *In-situ* AFM experiments were conducted on a JPK NanoWizard Ultra Speed II AFM fitted upon a Zeiss Axio Observer inverted optical microscope. All images were collected in tapping (AC) mode using a Nanoworld USC-F0.3-k0.3 cantilever with a nominal force constant of 0.3 $Nm^{-1}$ and at scan rates of 20 Hz and 30 Hz for the flexing expansion and flexing contraction transformations respectively. Samples were dispersed on a glass slide and held in place using a thin layer of Flexbar Reprorubber, two-part, thin pour adhesive. This adhesive was also used to adhere a custom-made, stainless-steel flow cell of volume 1 $cm^3$ to the glass slide. Fluid was introduced and

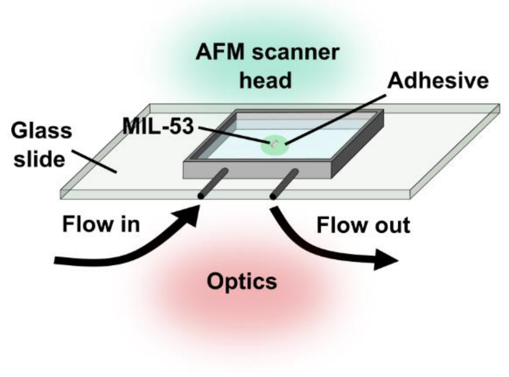


**Scheme S1:** Schematic representation depicting the fluid cell used for *in-situ* atomic force microscopy imaging.

removed from the flow cell using a Harvard Apparatus PHD 2000 syringe pump. This experimental setup is shown in Scheme 1.

During AFM imaging, crystals of **1** were observed using optical microscopy and selected only if the crystal showed clear faceting and elongation along one direction which was taken to be the <100> direction.

To image the flexing expansion transformation from **1**·0.96DMF to **1**·*x*EtOH, **1**·0.96DMF was adhered within the AFM flow cell along with 1 $cm^3$ DMF. Whilst imaging, ethanol was introduced into the flow cell at a rate of 2 $cm^3$ $hour^{-1}$ and liquid was removed from the cell at the same rate. For the flexing contraction transformation from **1**·*x*EtOH to **1**·0.96DMF, **1**·0.96DMF was adhered within the flow cell and 1 $cm^3$ ethanol was added resulting in an immediate transformation to **1**·*x*EtOH. *In-situ* AFM images were collected whilst a 20:80 v/v solution of DMF in ethanol was injected into the flow cell at 1 ml $hour^{-1}$ whilst liquid was removed at the same rate. The concentrations within the unit cell were determined using the equation:

$$V_{add} = V_{cell}(1 - \exp\left(\frac{-rT}{V_{cell}}\right))$$

where $V_{add}$ is the volume added to the flow cell, $V_{cell}$ is the volume of the cell, $r$ is the flow rate in and out of the cell and $T$ is the time from the start of the flow.

AFM image processing was carried out using the JPK SPM data processing software package, with height analysis conducted using the Gwyddion software package.[9] Prior to analysis of height data, a first order line-levelling treatment was applied followed by plane levelling which was applied across the largest available flat terrace. During measurement of terrace heights, line profiles were extracted parallel to <100> directions to minimise the height variation associated with ridges along the <100> directions. Where changes in lines profiles over time are discussed, series of profiles are taken between fixed points on the surface to minimise the influence of drift. Additional first-order line-levelling was applied across terraced regions of the extracted line profiles such that line profiles were flat either side of the terrace step. Measurement of surface angles was achieved through plane fitting of regions either side of domain interfaces on levelled height data. Normals of the fitted planes were computed using Gwyddion.[9] The surface angle was computed as $(180 - \theta)°$, where $\theta$ is the subtended angle between normals.

During processing of image series to produce videos, images were aligned using the SIFT plugin within the software ImageJ.[10] The image size in video SV1 is 6.0 x 6.0 $\mu m^2$ and the image size in video SV2 is 0.85 x 0.85 $\mu m^2$.

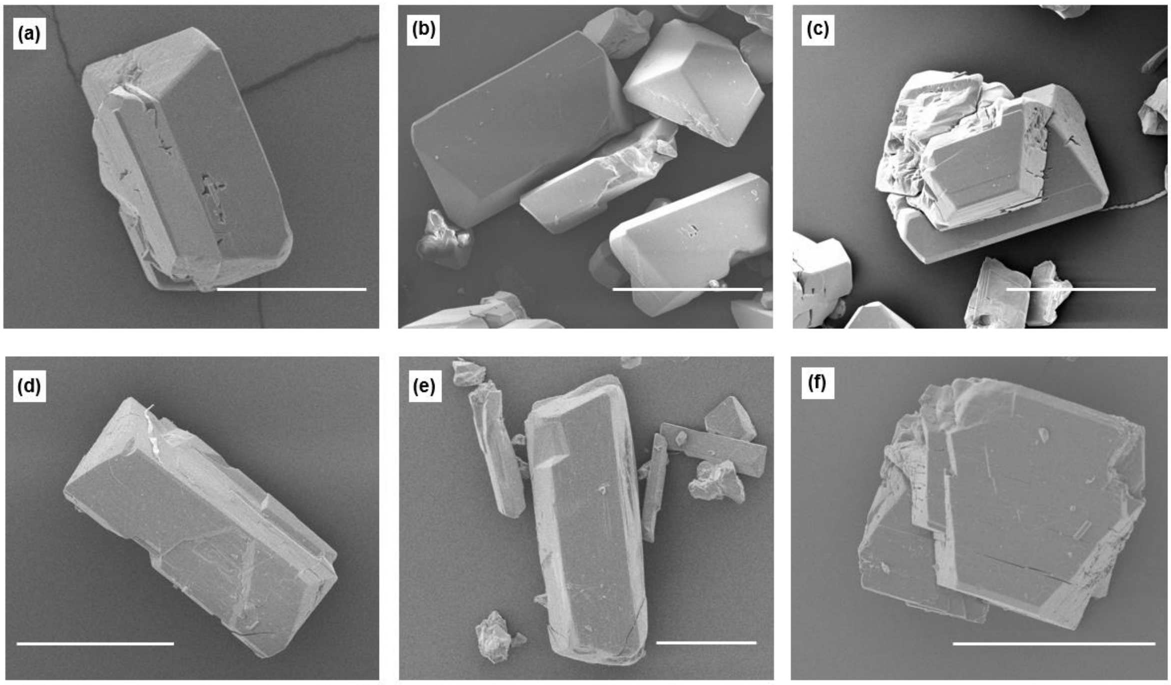


**Figure S1:** (a)-(c) Scanning electron micrographs of crystals of **1**·0.74$H_2$BDC. (d)-(f) Scanning electron micrographs of crystals of **1**·0.96DMF. Scale bars represent 100 μm.

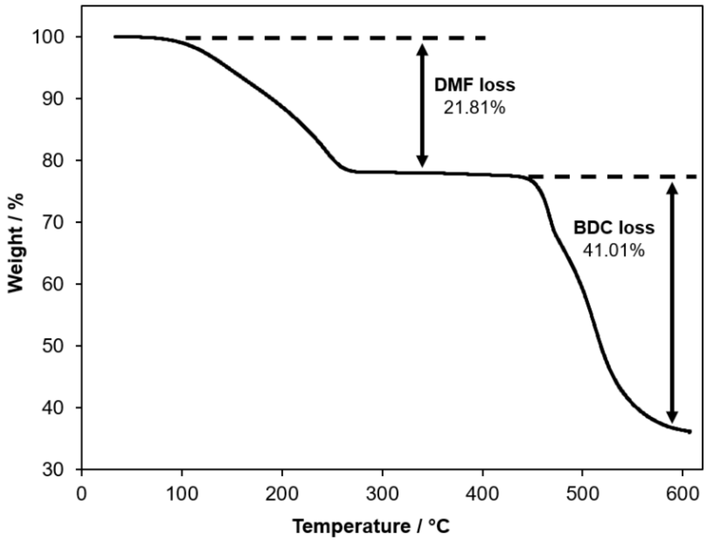


**Figure S2:** Thermograph of **1**·0.96DMF.

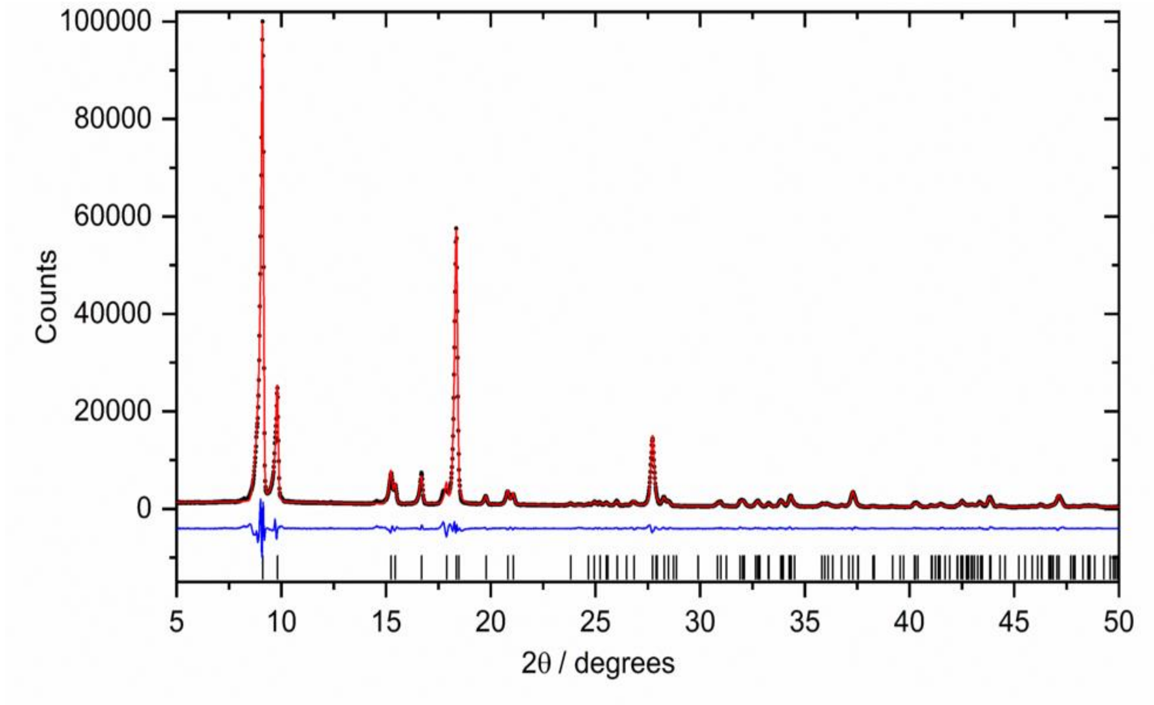


**Figure S3:** Le Bail fit for **1**·0.96DMF showing observed (dots), calculated (red) and difference (blue) plots (monoclinic, *I2/a*, $a$ = 6.7114(5) Å, $b$ = 11.4123(4) Å, $c$ = 17.89(1) Å, $\beta$ = 92.310(9)°, $V$ = 1368.8(1) Å$^3$, $R_{wp}$ = 8.21%, $R_p$ = 5.84 %). Reflection positions are shown as black ticks.

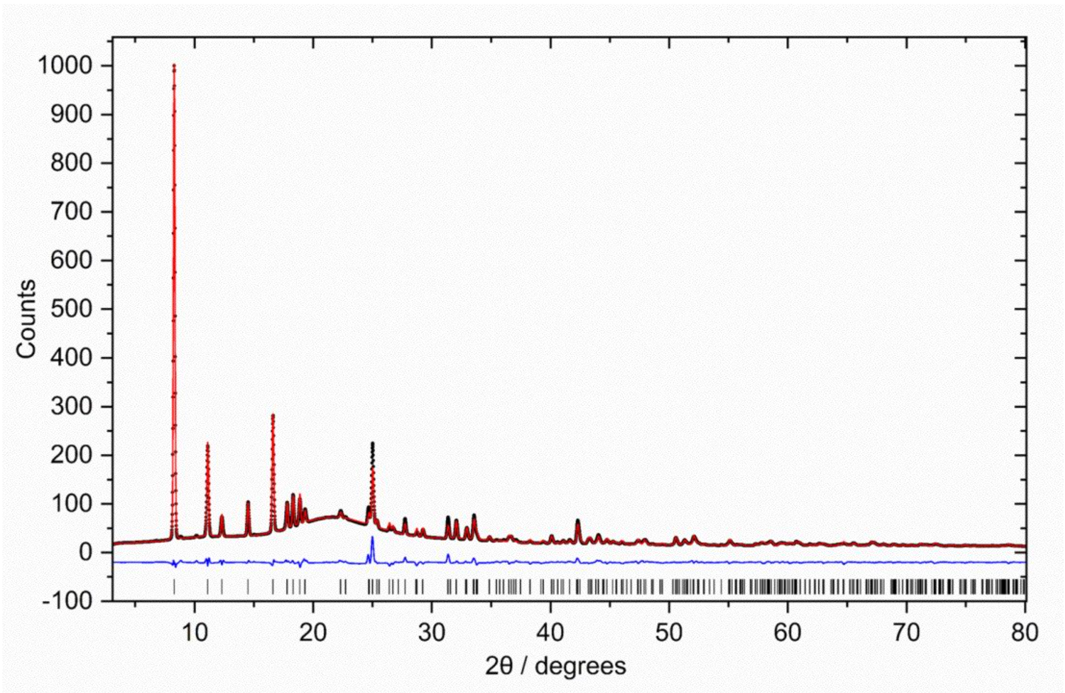


**Figure S4:** Final plot for Reitveld refinement for **1**·$x$EtOH showing observed (dots), calculated (red) and difference (blue) plots (orthorhombic, *Imcm*, $a$ = 6.7429(2) Å, $b$ = 14.3925(4) Å, $c$ = 15.9254(4) Å, $V$ = 1545.51(8) Å$^3$, $R_{wp}$ = 3.89 %, $R_p$ = 3.40 %). Reflection positions are shown as black ticks.

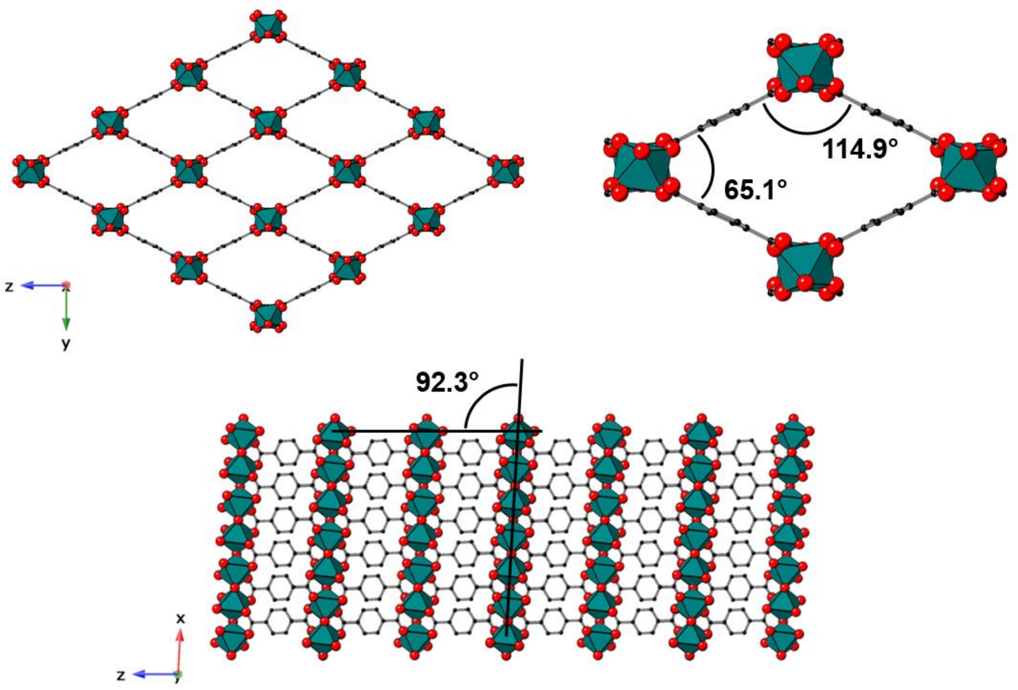


**Figure S5a:** The crystal structure of **1**·0.96DMF as reported by Chaplais *et al.* viewed down different crystallographic axes.[2] Colour key: Ga-centred octahedra = dark green; O atoms = red, C atoms = black. H atoms omitted for clarity. DMF guest molecule locations not determined.

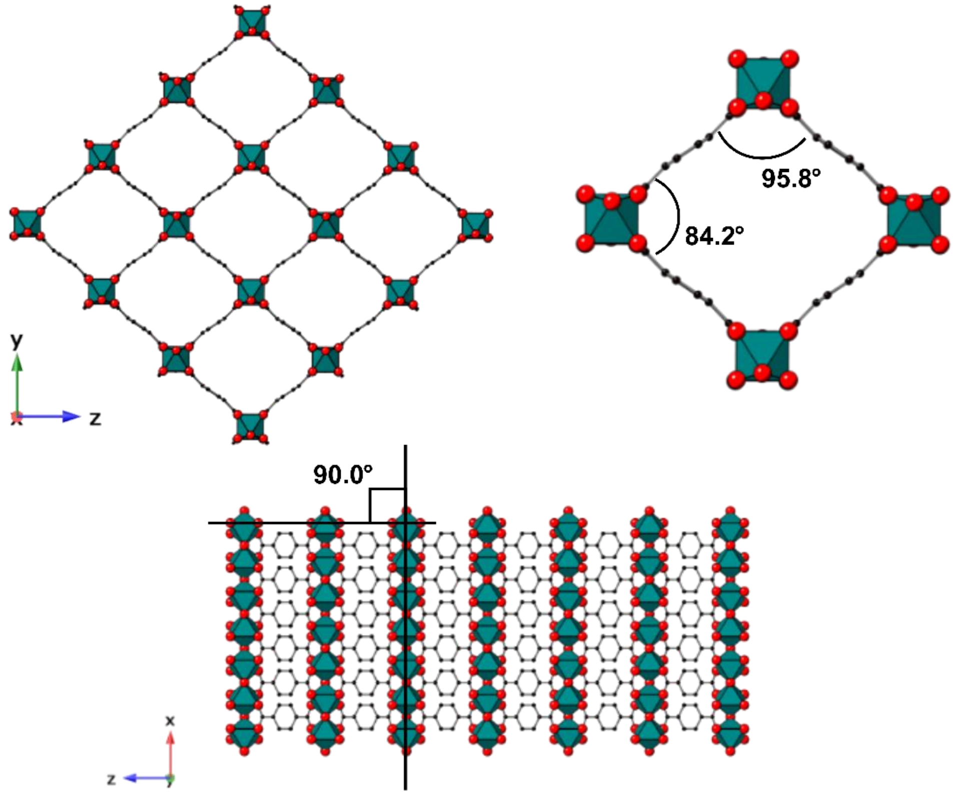


**Figure S5b:** The crystal structure of **1**·*x*EtOH viewed down different crystallographic axes. Colour key: Ga-centred octahedra = dark green; O atoms = red, C atoms = black. H atoms omitted for clarity. EtOH guest molecule locations not determined.

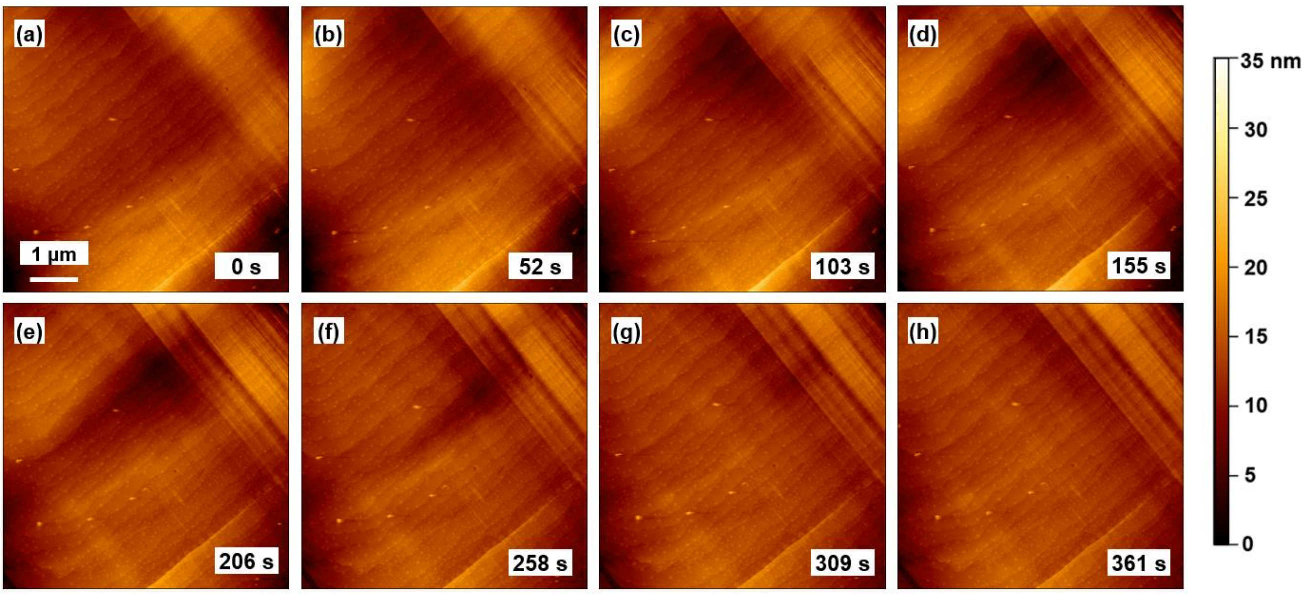


**Figure S6:** 6.0 x 6.0 µm AFM height images of a {011} facet of **1** during the pore expansion transformation from **1**·0.96DMF to **1**·xEtOH (see Figure 3 for error signal micrographs of the same image series).

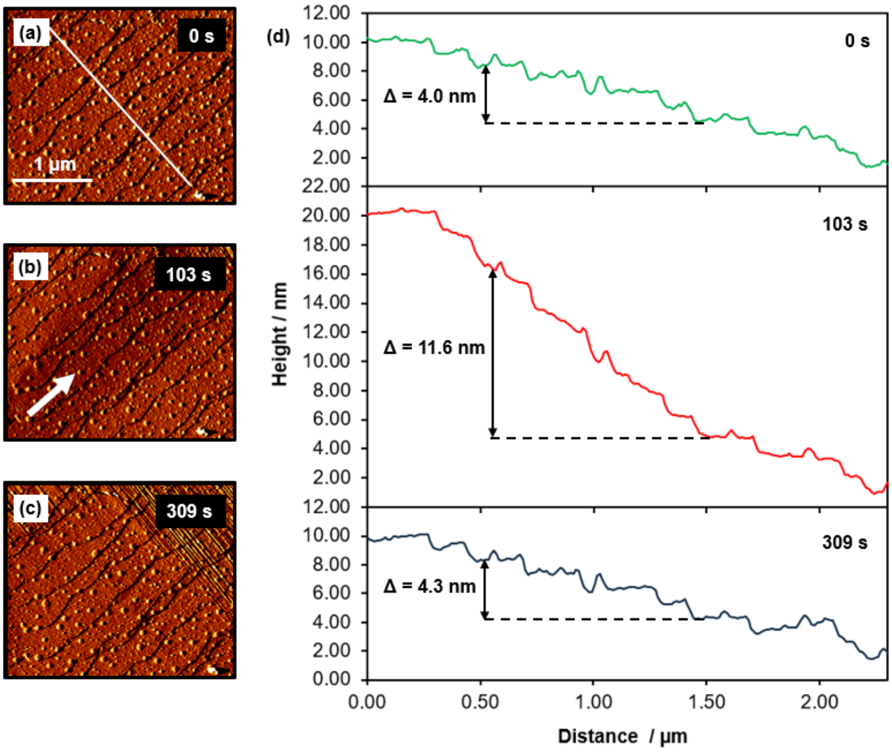


**Figure S7:** AFM error signal images of a {011} 2.86 x 2.53 µm region of **1**·0.96DMF prior to transformation (a, 0 s), during transformation to **1**·*x*EtOH (b, 103 s) and after transformation to **1**·*x*EtOH (c, 309 s) and the accompanying height profile of the white line shown in the AFM error signal image at 0 s at the corresponding times. The white arrow in (b) indicates the wavefront region.

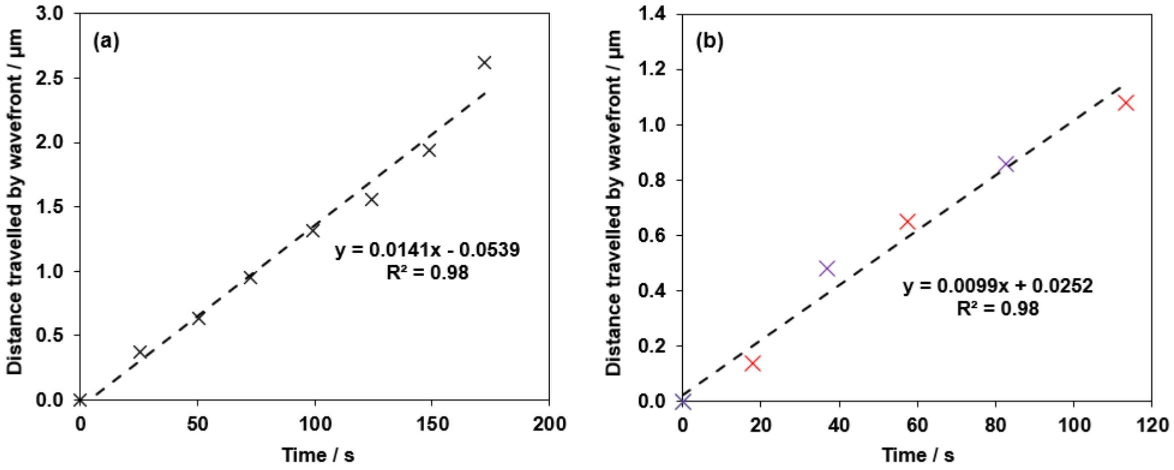


**Figure S8:** Plots of distance travelled by one wavefront (a) and two wavefronts (b) against time during the flexible expansion transformation (a) and the flexible contraction transformation (b).

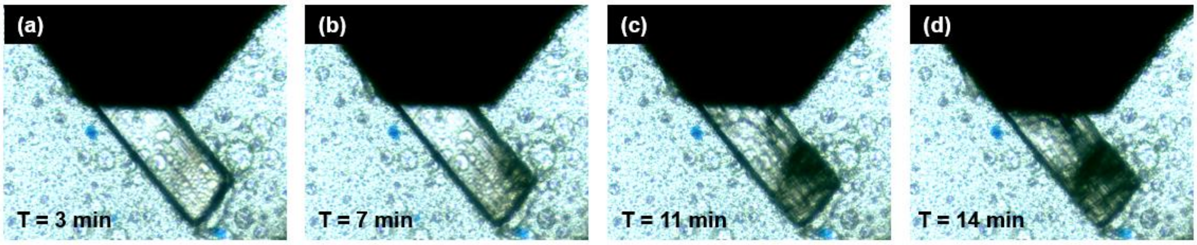


**Figure S9:** *In-situ* optical micrographs of a crystal of **1** taken during the flexing expansion transformation of **1**·0.96DMF to **1**·*x*EtOH showing a progressive loss of optical transparency during the transformation. Time stamps are given relative to the first changes observed in the AFM imaging.

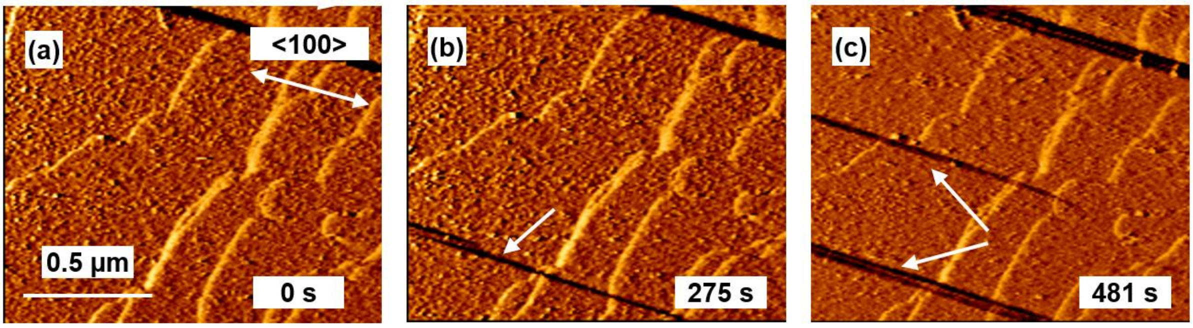


**Figure S10:** 1.5 x 1.2 µm AFM error signal images showing series of cracks developing along the <100> directions of **1** during transformation from **1**·0.96DMF to **1**·*x*EtOH. Time stamps are given relative to first observed change. These images were taken from a data set taken under equivalent conditions to those used for the data series shown in Figure 3.

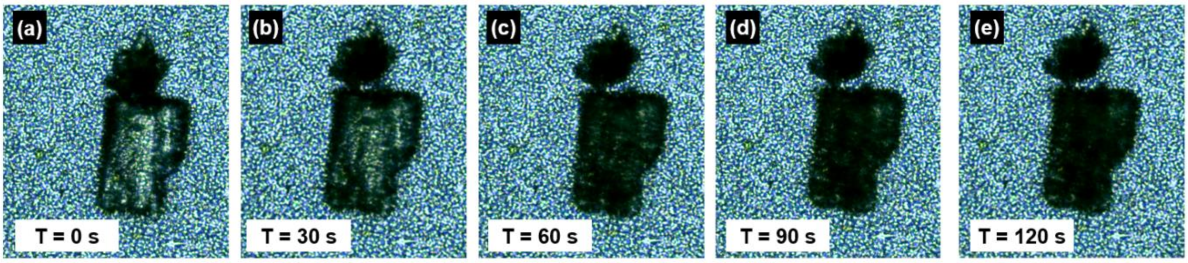


**Figure S11:** *In-situ* optical micrographs of crystals of **1** taken during the flexing expansion transformation of **1**·0.96DMF to **1**·*x*EtOH showing a rapid loss of optical transparency during the transformation.

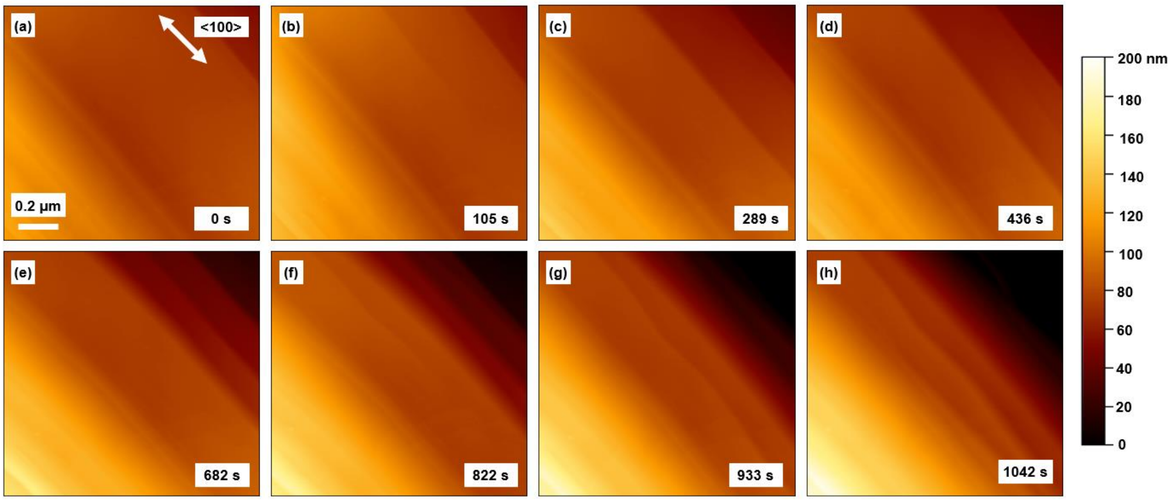


**Figure S12:** (a)-(h) An AFM height image series showing a 0.85 x 0.85 µm region of a {011} crystal surface during the flexing contraction transformation between **1**·*x*EtOH and **1**·0.96DMF. Time stamps are given relative to the first signs of change as observed under AFM imaging (see Figure 6 for error signal micrographs of the same image series).

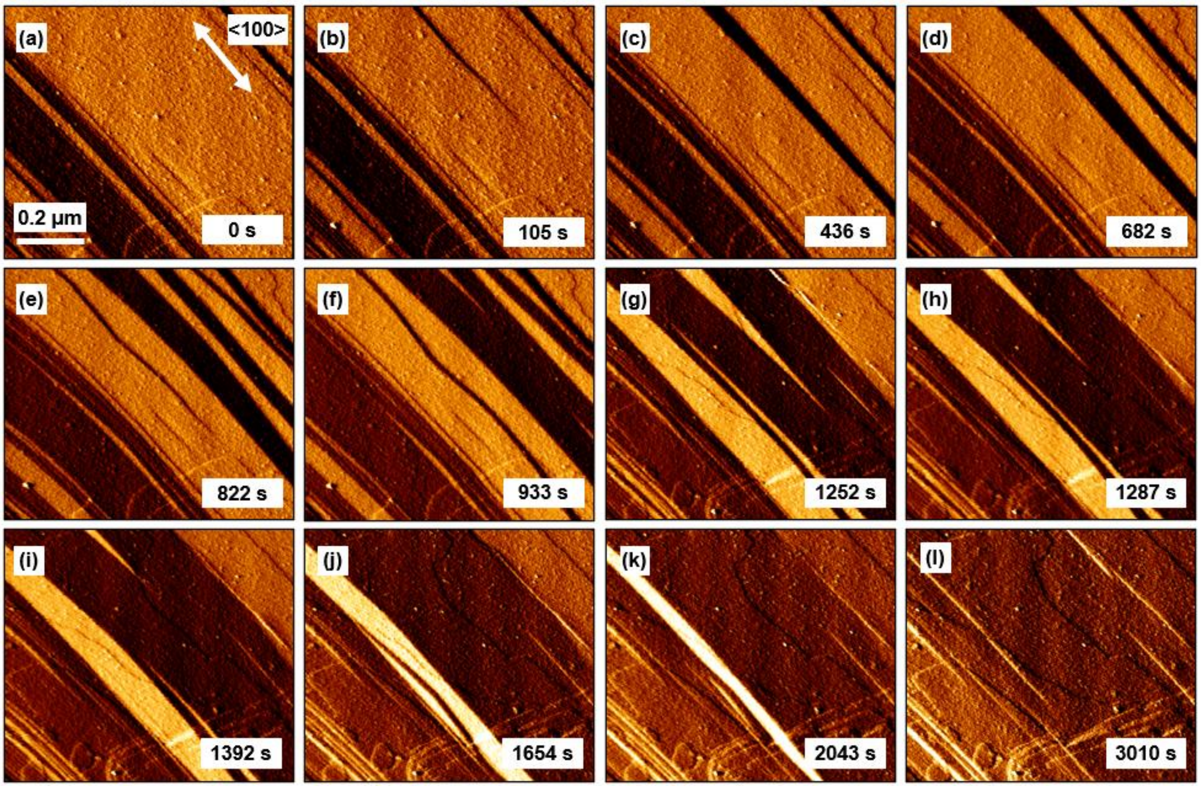


**Figure S13:** (a)-(l) An AFM error signal image series showing a 1.00 x 0.85 µm region of a {011} crystal surface during the flexing contraction transformation between **1**·*x*EtOH and **1**·0.96DMF. Time stamps are given relative to the first signs of change as observed under AFM imaging.

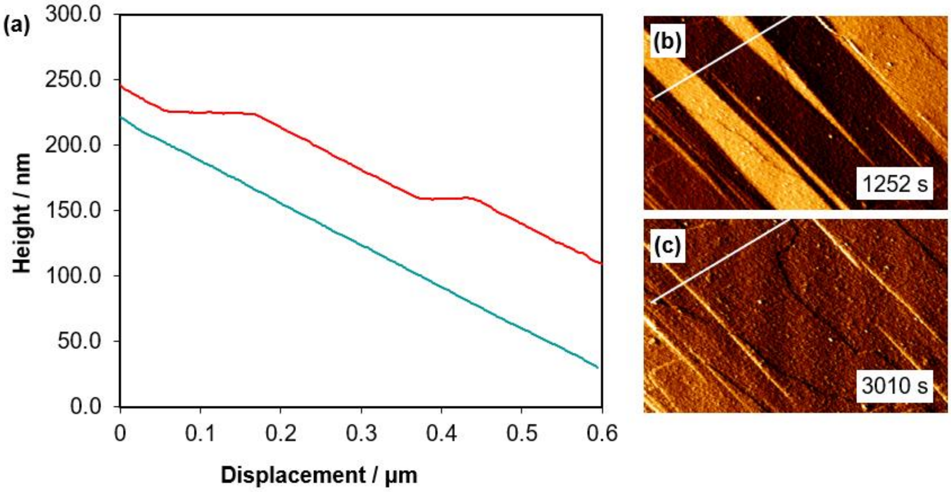


**Figure S14:** (a) Height profiles corresponding to the white lines shown in the 1.15 x 0.71 µm AFM error signal image (b) (red) and (c) (cyan) showing conversion of the angled {011} crystal surface (b) to a relatively smooth surface (c) during the flexing contraction transformation between **1**·*x*EtOH and **1**·0.96DMF. Time stamps are given relative to the first signs of change as observed under AFM imaging. (b) and (c) are the same images as those presented in Figure S13.

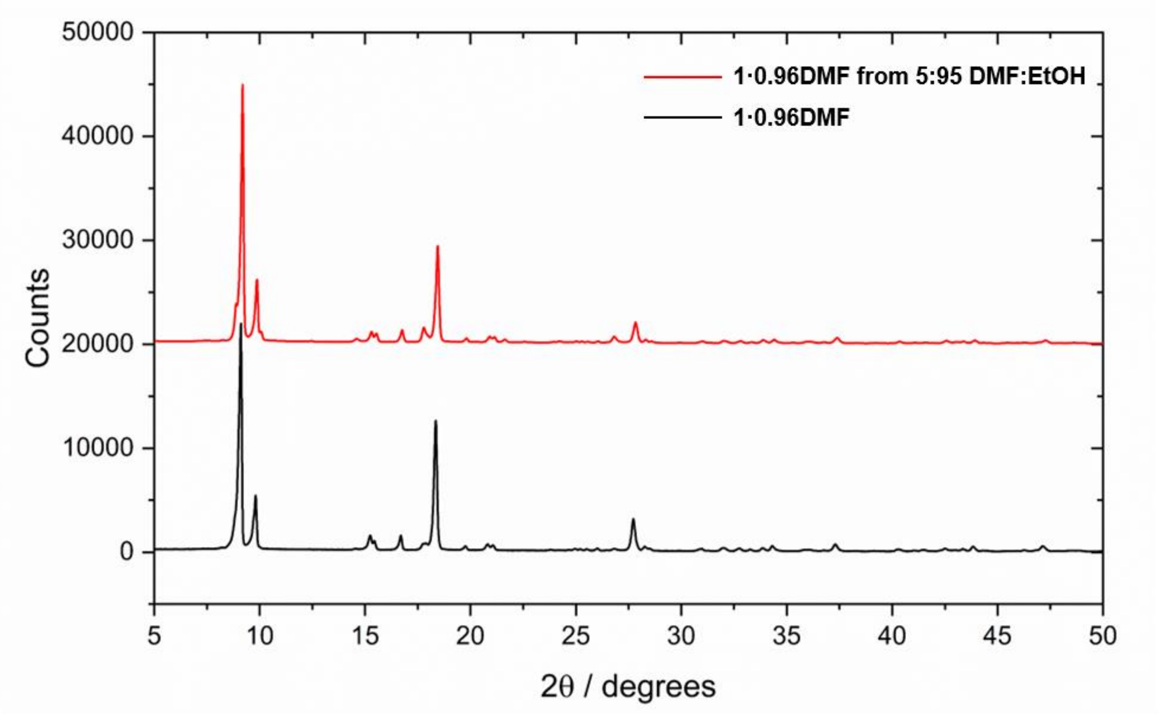


**Figure S15:** Comparison of the PXRD pattern of **1**·0.96DMF prior to any flexing transformation (black) and **1**·0.96DMF formed after flexing expansion and contraction transformations performed using the same transformation solution as that in the *in-situ* AFM flexing contraction study (red).

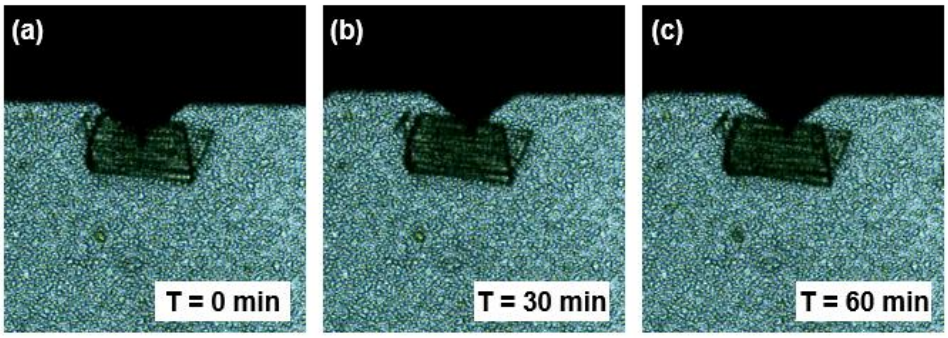


**Figure S16:** *In-situ* optical micrographs of crystals of **1** taken during the flexing contraction transformation of **1**·*x*EtOH to **1**·0.96DMF showing an approximately constant level of optical transparency during the transformation. Time stamps are shown relative to the first sign of transformation observed under AFM imaging.

**References:**


(1) M. Vougo-Zanda, J. Huang, E. Anokhina, X. Wang, A. J. Jacobson, *Inorg. Chem.* **2008**, *47*, 11535–11542.

(2) G. Chaplais, A. Simon-Masseron, F. Porcher, C. Lecomte, D. Bazer-Bachi, N. Bats, J. Patarin, *Phys. Chem. Chem. Phys.* **2009**, *11*, 5241–5245.

(3) O. V. Dolomanov, L. J. Bourhis, R. J. Gildea, J. A. K. Howard, H. Puschmann, *J. Appl. Crystallogr.* **2009**, *42*, 339–341.

(4) G. M. Sheldrick, *Acta Crystallogr. Sect. A* Found. Adv. **2015**, 71, 3–8.

(5) G. M. Sheldrick, *Acta Crystallogr. Sect. C* Struct. Chem. **2015**, 71, 3–8.

(6) A. R. B. J. Lutton-Gething, L. T. Nangkam, J. O. W. Johansson, I. Pallikara, J. M. Skelton, G. F. S. Whitehead, I. J. Vitorica-Yrezabal, M. P. Attfield, *Chem. Eur. J.* **2023**, *29*, e202203773.

(7) R. I. Walton, A. S. Munn, N. Guillou, F. Millange, *Chem. Eur. J.* **2011**, *17*, 7069–7079.

(8) B. H. Toby, R. B. von Dreele, *J. Appl. Cryst.* **2013**, *46*, 544-549.

(9) D. Nečas, P. Klapetek, *Open Phys.* **2012**, *10*, 181–188.

(10) D. G. Lowe, *Int. J. Comput. Vis.* **2004**, *60*, 91–110.
